# Role of Inositol Hexakisphosphate Kinases in Vascular Smooth Muscle Cell Calcification

**DOI:** 10.3390/ijms27031411

**Published:** 2026-01-30

**Authors:** Sheyda Bahiraii, Isratul Jannat, Sarah Plösser, Mehdi Razazian, Jakob Voelkl, Ioana Alesutan

**Affiliations:** 1 Institute for Physiology and Pathophysiology, Johannes Kepler University Linz, Altenbergerstrasse 69, 4040 Linz, Austria; 2 Department of Nephrology and Medical Intensive Care, Charité-Universitätsmedizin Berlin, Corporate Member of Freie Universität Berlin and Humboldt Universität zu Berlin, Augustenburger Platz 1, 13353 Berlin, Germany; 3 DZHK (German Centre for Cardiovascular Research), Partner Site Berlin, 13347 Berlin, Germany

**Keywords:** chronic kidney disease, phosphate, vascular calcification, vascular smooth muscle cells, inositol hexakisphosphate kinase, IP6K, AKT, SGK1, 5-IP7

## Abstract

Phosphate-induced vascular calcification in chronic kidney disease is linked to cardiovascular mortality. This calcification process involves vascular smooth muscle cells (VSMCs), which can promote a pro-calcific environment in the vascular wall. However, the mechanisms underlying a putative phosphate sensing of VSMCs to modulate pro-calcific signaling are insufficiently clarified. In mammals, three isoforms of the inositol hexakisphosphate kinase (IP6K) exist, which have been implicated in cellular phosphate homeostasis. Therefore, each IP6K isoform was silenced in calcifying primary human aortic VSMCs. *IP6K1* and *IP6K2* mRNA expression were increased in calcifying VSMCs. Silencing of either IP6K1 or IP6K2 ameliorated phosphate-induced pro-calcific markers expression and VSMC calcification. *IP6K3* mRNA expression was not modified during calcifying conditions, but IP6K3 silencing still resulted in some anti-calcific effects. Mechanistically, the IP6K product 5-IP7 may act as a potent inhibitor of AKT kinase signaling. Accordingly, pro-calcific conditions induced only transient AKT phosphorylation, and IP6K2 silencing increased AKT phosphorylation in calcifying VSMCs. In turn, AKT inhibition blunted the protective effects of IP6K2 knockdown, while serum- and glucocorticoid-inducible kinase 1 (SGK1) inhibition restored these effects. These observations indicate a role for IP6Ks during phosphate-induced VSMC calcification, which could be mediated by an altered balance between AKT and SGK1 signaling.

## 1. Introduction

Medial vascular calcification (VC) is common in patients with chronic kidney disease (CKD) [1,2] and is linked to death and hospitalization in these patients [3]. Progression of VC especially increases in late stages of CKD [4], where serum phosphate levels also rise [5].

Phosphate circulating in serum harbors the risk of extraosseous calcium-phosphate complexation [6]. Under physiological conditions, the extraosseous growth of calcium-phosphate crystals is prevented by an endogenous mineral buffering system [6]. This consists of multiple known and unknown components, such as Fetuin-A, matrix GLA protein, pyrophosphate, magnesium, or zinc [7,8,9,10,11]. In CKD, the endogenous anti-calcific mechanisms are insufficient [10], and phosphate has emerged as a key promoter of VC [2].

Phosphate induces alterations in vascular smooth muscle cells (VSMCs), involving inflammatory and senescent pathways that ultimately induce a modulation of vascular mineralization processes [12,13,14]. Phosphate-stressed VSMCs also produce pro-calcific signaling molecules, such as osteocalcin (also known as bone gamma-carboxyglutamate protein or BGLAP) [15] or bone morphogenic protein 2 (BMP2) [16]. These signaling molecules induce multiple and pro-inflammatory pathways in VSMCs, thereby shifting the balance to a pro-calcific microenvironment [8]. The pro-calcific effects mediated by VSMCs involve the release of extracellular vesicles, alteration of the extracellular matrix, and reduction of endogenous calcification inhibitors [8].

The re-programming of VSMCs towards a pro-calcific phenotype is regulated by complex signaling pathways, where especially the transcription factor core-binding factor alpha 1 (CBFA1, also termed runt-related transcription factor 2 or RUNX2) is considered an integrating master-regulator of VC [12,17]. While VSMCs can generally inhibit ectopic deposition of calcium and phosphate by producing pyrophosphate, phosphate-stressed VSMCs increase the production of tissue-nonspecific alkaline phosphatase (ALPL) [18]. ALPL cleaves the calcification inhibitor pyrophosphate into two inorganic phosphate molecules, which leads to a loss of inhibitory capacity on VC [7].

However, the exact mechanism by which phosphate exposure affects VSMC function is incompletely understood. Phosphate may be taken up by VSMCs through direct transport mechanisms [19] or in the form of calciprotein particles by endocytosis [10]. One possible pathway to regulate intracellular phosphate homeostasis is through inositol pyrophosphate produced by inositol hexakisphosphate kinases (IP6Ks) [20]. IP6Ks can phosphorylate inositol hexakisphosphate (IP6) to form 5-diphosphoinositol pentakisphosphate (5-IP7) [21]. IP6Ks are present in three isoforms, of which IP6K1 and IP6K2 are more ubiquitously expressed [22]. IP6K3 is expressed in a more tissue-specific manner, with expression more strongly detected in muscular and brain tissues [22]. IP6Ks can regulate cellular phosphate export, and IP6K1/2-knockout cells exhibit increased intracellular phosphate levels [20]. IP6K1/2 deficiency in tubular cells disturbs systemic phosphate homeostasis in mice, where at least male mice develop hypophosphatemia [23]. Pharmacological inhibition of IP6K reduces circulating phosphate levels and VC in rats after adenine-induced CKD [24]. In addition, the inositol pyrophosphate 5-IP7 also acts as a physiological inhibitor of AKT kinase, linking IP6K to insulin sensitivity [25], which, in turn, could be involved in VSMC calcification [26].

Therefore, the function of IP6K in VSMCs during phosphate exposure may be important in VSMC calcification. This study systematically investigated the effects of isoform-specific gene silencing of IP6K in phosphate-treated VSMCs.

## 2. Results

**Figure 1:**
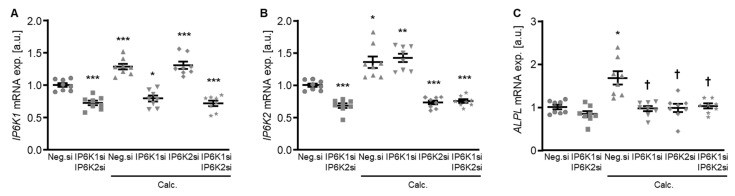
Effects of IP6K1 and IP6K2 single or double knockdown on pro-calcific marker expression in VSMCs during calcifying conditions. Relative mRNA expression (*n* = 8) of *IP6K1* (**A**), *IP6K2* (**B**), and *ALPL* (**C**) in HAoSMCs transfected for 72 h with negative control (Neg.si), IP6K1 (IP6K1si), and/or IP6K2 (IP6K2si) siRNA and treated for 48 h with control or calcification medium (Calc.). * (*p* < 0.05), ** (*p* < 0.01), *** (*p* < 0.001): significant compared to Neg.si-transfected group; † (*p* < 0.05): significant compared to Neg.si-transfected and Calc.-treated group.

To explore a possible impact of the IP6Ks on VSMC calcification, experiments were performed by suppressing their endogenous expression in primary human aortic smooth muscle cells (HAoSMCs) by silencing the respective genes using small interfering RNA (siRNA) during control and calcifying conditions with high phosphate and calcium levels (calcification medium). Experiments with double knockdown of the two dominant IP6K isoforms, IP6K1 and IP6K2, in HAoSMCs showed an increased mRNA expression of both isoforms during calcifying conditions (Figure 1A,B). Both individual and simultaneous knockdown of IP6K1 and IP6K2 blunted the calcification medium-induced *ALPL* mRNA expression in HAoSMCs (Figure 1C).

**Figure 2:**
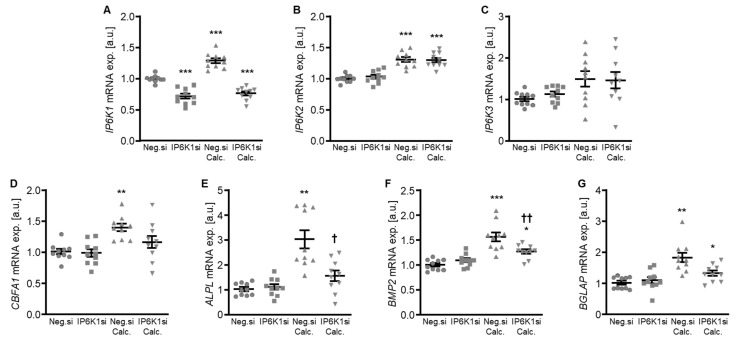
Effects of IP6K1 knockdown on IP6K isoform and pro-calcific marker expression in VSMCs during calcifying conditions. Relative mRNA expression (*n* = 10) of *IP6K1* (**A**), *IP6K2* (**B**), *IP6K3* (**C**), *CBFA1* (**D**), *ALPL* (**E**), *BMP2* (**F**), and *BGLAP* (**G**) in HAoSMCs transfected for 72 h with negative control (Neg.si) or IP6K1 (IP6K1si) siRNA and treated for 48 h with control or calcification medium (Calc.). * (*p* < 0.05), ** (*p* < 0.01), *** (*p* < 0.001): significant compared to Neg.si-transfected group; † (*p* < 0.05), †† (*p* < 0.01): significant compared to Neg.si-transfected and Calc.-treated group.

**Figure 3:**
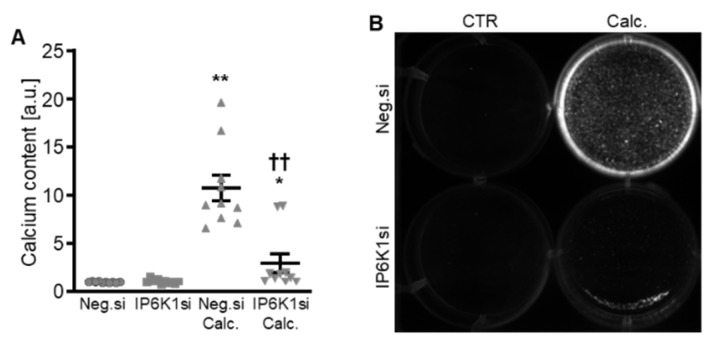
Effects of IP6K1 knockdown on VSMC calcification during calcifying conditions. Normalized calcium content (*n* = 10, **A**) and calcification detected by Osteosense fluorescence imaging (**B**) in HAoSMCs transfected with negative control (Neg.si) or IP6K1 (IP6K1si) siRNA and treated for 11d with control (CTR) or calcification medium (Calc.). Calcified areas: white pseudocolor. * (*p* < 0.05), ** (*p* < 0.01): significant compared to Neg.si-transfected group; †† (*p* < 0.01): significant compared to Neg.si-transfected and Calc.-treated group.

Therefore, the silencing of IP6K isoforms during calcifying conditions was investigated in detail. Calcification medium significantly increased *IP6K1* and *IP6K2* mRNA expression, but did not significantly modify *IP6K3* mRNA expression (*p* = 0.118) in negative control siRNA-transfected HAoSMCs (Figure 2A–C). Transfection with IP6K1 siRNA significantly reduced *IP6K1* mRNA expression in both control- and calcification medium-treated HAoSMCs (Figure 2A). Silencing of the IP6K1 isoform did not significantly affect *IP6K2* and *IP6K3* isoforms mRNA expression (Figure 2B,C). Calcification medium significantly up-regulated the mRNA expression of the pro-calcific markers *CBFA1*, *ALPL*, *BMP2,* and *BGLAP* (Figure 2D–G). Knockdown of IP6K1 did not significantly affect the pro-calcific marker mRNA expression during control conditions, but significantly suppressed *ALPL* and *BMP2* mRNA expression and tended to reduce *CBFA1* and *BGLAP* mRNA expression during calcifying conditions; these differences, however, did not reach statistical significance (*p* = 0.084 and *p* = 0.067, respectively). More importantly, silencing of the IP6K1 gene significantly reduced calcification of HAoSMCs, as determined by quantification of the calcium content and Osteosense staining (Figure 3). Thus, knockdown of the IP6K1 isoform reduced pro-calcific signaling and calcification of HAoSMCs during calcifying conditions.

**Figure 4:**
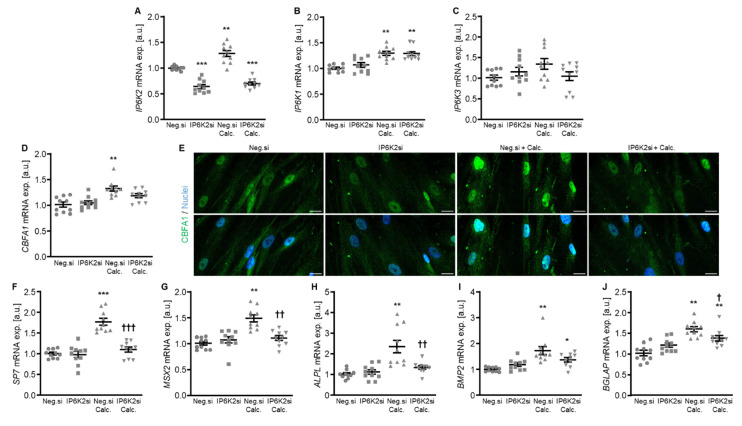
Effects of IP6K2 knockdown on IP6K isoform and pro-calcific marker expression in VSMCs during calcifying conditions. Relative mRNA expression (*n* = 10) of *IP6K2* (**A**), *IP6K1* (**B**), *IP6K3* (**C**), *CBFA1* (**D**), *SP7* (**F**), *MSX2* (**G**), *ALPL* (**H**), *BMP2,* (**I**) and *BGLAP* (**J**) in HAoSMCs transfected for 72h with negative control (Neg.si) or IP6K2 (IP6K2si) siRNA and treated for 48 h with control or calcification medium (Calc.). * (*p* < 0.05), ** (*p* < 0.01), *** (*p* < 0.001): significant compared to Neg.si-transfected group; † (*p* < 0.05), †† (*p* < 0.01), ††† (*p* < 0.001): significant compared to Neg.si-transfected and Calc.-treated group. (**E**) CBFA1 (green) and nuclei (blue) shown by confocal imaging in HAoSMCs transfected for 72 h with negative control (Neg.si) or IP6K2 (IP6K2si) siRNA and treated for 48 h with control or calcification medium (Calc.). Scale bar: 20 µm.

**Figure 5:**
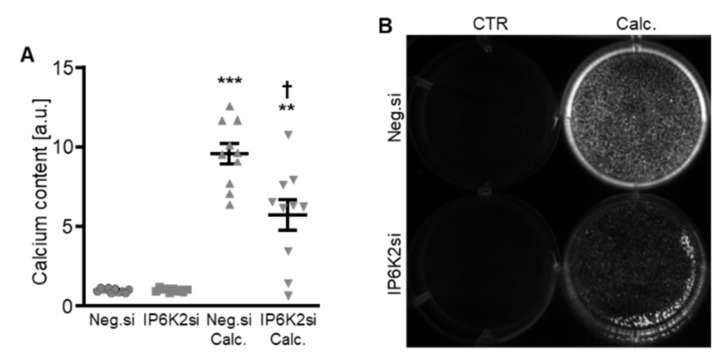
Effects of IP6K2 knockdown on VSMC calcification during calcifying conditions. Normalized calcium content (*n* = 10, **A**) and calcification detected by Osteosense fluorescence imaging (**B**) in HAoSMCs transfected with negative control (Neg.si) or IP6K2 (IP6K2si) siRNA and treated for 11d with control (CTR) or calcification medium (Calc.). Calcified areas: white pseudocolor. ** (*p* < 0.01), *** (*p* < 0.001): significant compared to Neg.si-transfected group; † (*p* < 0.05): significant compared to Neg.si-transfected and Calc.-treated group.

Additional experiments focused on a more in-depth characterization of IP6K2 silencing, as this might be the most abundant IP6K isoform in human aorta [22]. As shown in Figure 4A, *IP6K2* mRNA expression was significantly reduced following transfection with IP6K2 siRNA during both control and calcifying conditions. Silencing of IP6K2 did not significantly change the mRNA expression of the *IP6K1* and *IP6K3* isoforms (Figure 4B,C). IP6K2 knockdown blunted the calcification medium-induced *SP7* (encoding Osterix), *MSX2*, *ALPL,* and *BGLAP* mRNA expression as well as CBFA1 nuclear localization in HAoSMCs, but did not significantly modify calcification medium-induced *CBFA1* and *BMP2* mRNA expression (*p* = 0.249 and *p* = 0.285, respectively) (Figure 4D–J). Moreover, silencing of the IP6K2 gene significantly suppressed calcification of HAoSMCs induced by exposure to calcification medium (Figure 5). During control conditions, IP6K2 knockdown did not significantly modify pro-calcific marker expression or calcification of HAoSMCs (Figure 4D–J and Figure 5). Taken together, knockdown of the IP6K2 isoform reduced some aspects of pro-calcific signaling and calcification of HAoSMCs during calcifying conditions.

**Figure 6:**
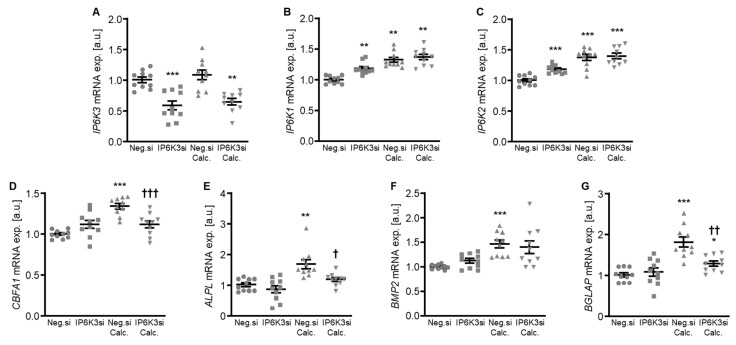
Effects of IP6K3 knockdown on IP6K isoform and pro-calcific marker expression in VSMCs during calcifying conditions. Relative mRNA expression (*n* = 10) of *IP6K3* (**A**), *IP6K1* (**B**), *IP6K2* (**C**), *CBFA1* (**D**), *ALPL* (**E**), *BMP2,* (**F**) and *BGLAP* (**G**) in HAoSMCs transfected for 72 h with negative control (Neg.si) or IP6K3 (IP6K3si) siRNA and treated for 48 h with control or calcification medium (Calc.). * (*p* < 0.05), ** (*p* < 0.01), *** (*p* < 0.001): significant compared to Neg.si-transfected group; † (*p* < 0.05), †† (*p* < 0.01), ††† (*p* < 0.001): significant compared to Neg.si-transfected and Calc.-treated group.

**Figure 7:**
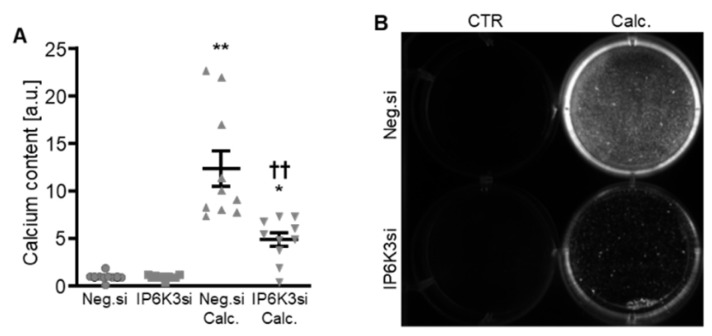
Effects of IP6K3 knockdown on VSMC calcification during calcifying conditions. Normalized calcium content (*n* = 10, **A**) and calcification detected by Osteosense fluorescence imaging (**B**) in HAoSMCs transfected with negative control (Neg.si) or IP6K3 (IP6K3si) siRNA and treated for 11d with control (CTR) or calcification medium (Calc.). Calcified areas: white pseudocolor. * (*p* < 0.05), ** (*p* < 0.01): significant compared to Neg.si-transfected group; †† (*p* < 0.01): significant compared to Neg.si-transfected and Calc.-treated group.

To systematically complement the experiments, IP6K3 silencing was also investigated. Silencing of the IP6K3 gene significantly downregulated *IP6K3* mRNA expression in HAoSMCs (Figure 6A) and, surprisingly, significantly up-regulated the mRNA expression of the other two isoforms, *IP6K1* and *IP6K2,* in control-treated HAoSMCs (Figure 6B,C). Knockdown of IP6K3 significantly suppressed calcification medium-induced *CBFA1*, *ALPL* and *BGLAP* mRNA expression, without significantly affecting calcification medium-induced *BMP2* mRNA expression (*p* = 0.974) (Figure 6D–G). Furthermore, IP6K3 knockdown significantly reduced calcification of HAoSMCs during calcifying conditions (Figure 7). Again, silencing of the IP6K3 gene alone did not significantly modify pro-calcific marker expression or calcification of HAoSMCs (Figure 6D–G and Figure 7). Thus, knockdown of the IP6K3 isoform also reduced pro-calcific signaling and calcification of HAoSMCs during calcifying conditions.

**Figure 8:**
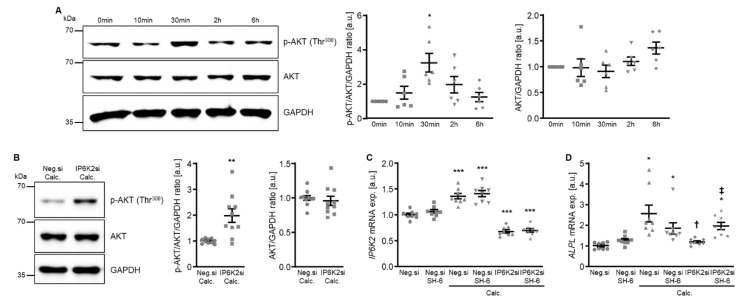
Involvement of AKT signaling in the protective effects of IP6K2 knockdown in VSMCs during calcifying conditions. (**A**) Representative Western blots and normalized phospho-AKT (Thr^308^) and total AKT protein abundance (*n* = 6) in HAoSMCs treated for the indicated time (0–6 h) with calcification medium. (**B**) Representative Western blots and normalized phospho-AKT (Thr^308^) and total AKT protein abundance (*n* = 10) in HAoSMCs transfected for 48 h with negative control (Neg.si) or IP6K2 (IP6K2si) siRNA and treated for 6 h with calcification medium (Calc.). Relative mRNA expression (*n* = 8) of *IP6K2* (**C**) and *ALPL* (**D**) in HAoSMCs transfected for 72 h with negative control (Neg.si) or IP6K2 (IP6K2si) siRNA and treated for 48 h with control or calcification medium (Calc.) without and with 10 µM AKT inhibitor SH-6. * (*p* < 0.05), ** (*p* < 0.01), *** (*p* < 0.001): significant compared to control/Neg.si-transfected+Calc.-treated/Neg.si-transfected group; † (*p* < 0.05): significant compared to Neg.si-transfected and Calc.-treated group; ‡ (*p* < 0.05): significant between IP6K2si-transfected and Calc.- or Calc.+SH-6-treated groups.

To explore the potential underlying mechanisms of the protective effects of IP6K knockdown in HAoSMCs during calcifying conditions, additional experiments investigated the potential involvement of AKT signaling. As shown by Western blotting, calcification medium significantly increased AKT phosphorylation at Thr^308^ following 30 min of exposure. This increased AKT phosphorylation appeared transient and was not observed at later time points of 2 h and 6 h (Figure 8A). However, silencing of IP6K2 led to a significantly higher AKT phosphorylation in HAoSMCs exposed for 6 h to calcification medium (Figure 8B). To further explore a potential role of increased AKT activation in the protective effects of IP6K2 knockdown in HAoSMCs, the effects of pharmacological AKT inhibition using SH-6 during calcifying conditions were determined. As shown in Figure 8C, *IP6K2* mRNA expression was significantly reduced in IP6K2 siRNA-transfected as compared to negative control siRNA-transfected HAoSMCs, while additional treatment with the SH-6 inhibitor did not significantly affect *IP6K2* mRNA expression, either during control or calcifying conditions. Moreover, co-treatment with SH-6 did not significantly affect *ALPL* mRNA expression in negative control siRNA-transfected HAoSMCs exposed to control or calcification medium, but significantly blunted the inhibitory effects of IP6K2 silencing on calcification medium-induced *ALPL* mRNA expression (Figure 8D). Thus, AKT activation may be involved in the protective effects of IP6K knockdown during HAoSMCs calcification.

To further explore how IP6K2 knockdown-induced AKT activation during calcifying conditions may interfere with pro-calcific signaling in HAoSMCs, the effects on potential downstream signaling were determined. As a result, silencing of IP6K2 did not significantly modify calcification medium-induced mRNA expression of the phosphate transporters: xenotropic and polytropic retrovirus receptor 1 (*XPR1*) and solute carrier family 20 member 1 (*SLC20A1*, also known as sodium-dependent phosphate cotransporter 1 or *PiT1*), while AKT inhibition with SH-6 significantly up-regulated *XPR1* mRNA expression and tended to up-regulate *SLC20A1* mRNA expression (*p* = 0.169) in HAoSMCs during control conditions, but not during calcifying conditions without or with IP6K2 knockdown (Supplementary Figure S1).

**Figure 9:**
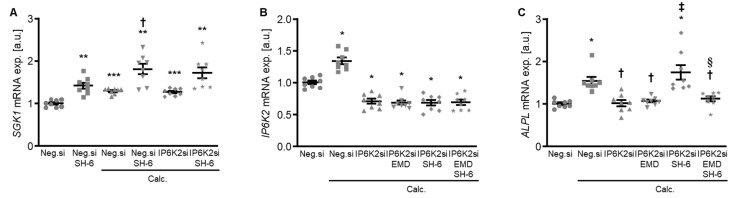
Role of AKT and SGK1 signaling interplay in the protective effects of IP6K2 knockdown in VSMCs during calcifying conditions. (**A**) Relative mRNA expression (*n* = 8) of *SGK1* in HAoSMCs transfected for 72 h with negative control (Neg.si) or IP6K2 (IP6K2si) siRNA and treated for 48h with control or calcification medium (Calc.) without and with 10 µM AKT inhibitor SH-6. Relative mRNA expression (*n* = 8) of *IP6K2* (**B**) and *ALPL* (**C**) in HAoSMCs transfected for 72 h with negative control (Neg.si) or IP6K2 (IP6K2si) siRNA and treated for 48 h with control or calcification medium (Calc.) without and with 50 µM SGK1 inhibitor EMD638683 (EMD) and/or 10 µM AKT inhibitor SH-6. * (*p* < 0.05), ** (*p* < 0.01), *** (*p* < 0.001): significant compared to Neg.si-transfected group; † (*p* < 0.05): significant compared to Neg.si-transfected and Calc.-treated group; ‡ (*p* < 0.05): significant compared to IP6K2si-transfected and Calc.-treated group; § (*p* < 0.05): significant between IP6K2si-transfected and Calc.+SH-6- or Calc.+SH-6+EMD-treated groups.

Moreover, treatment with the AKT inhibitor SH-6 significantly increased the mRNA expression of serum- and glucocorticoid-inducible kinase 1 (*SGK1*) in HAoSMCs during control and calcifying conditions (Figure 9A), a critical pro-calcific factor in VC [27]. In addition, pharmacological inhibition of SGK1 with EMD638683 restored the protective effects of IP6K2 knockdown during AKT inhibition on the pro-calcific signaling in HAoSMCs (Figure 9B,C). Thus, an interplay between AKT and SGK1 signaling may be involved in the protective effects of IP6K2 knockdown in HAoSMCs during calcifying conditions.

## 3. Discussion

**Figure 10:**
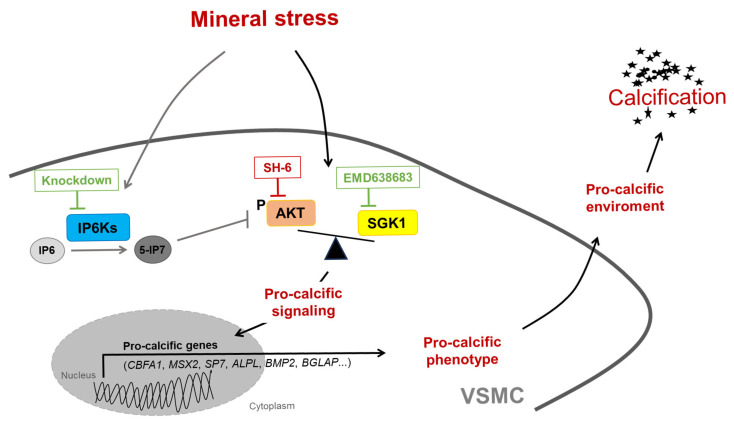
Schematic illustration of the potential role of IP6Ks during VSMC calcification. Disbalanced phosphate and calcium homeostasis leads to mineral stress, which may trigger activation of pro-calcific signaling in vascular smooth muscle cells (VSMCs), causing vascular calcification. Mineral stress also increases the expression of inositol hexakisphosphate kinases (IP6Ks) in VSMCs. IP6Ks mediate the conversion of inositol hexakisphosphate (IP6) into 5-diphosphoinositol pentakisphosphate (5-IP7), a potent inhibitor of AKT signaling. Knockdown of any of the three IP6K isoforms inhibits pro-calcific signaling and calcification of VSMCs during calcifying conditions. A key role may be attributed to the interplay between AKT and SGK1 signaling, as pharmacological inhibition of AKT (SH-6) could induce a shift towards the pro-calcific SGK1 signaling and abolishment of the anti-calcific effects of IP6K knockdown, while additional inhibition of SGK1 (EMD638683) restores these protective effects. However, other mechanisms not depicted here may be involved, and further evidence is required to support this hypothesis.

The current results indicate a pro-calcific effect of IP6K in VSMCs, as gene silencing of any of the three IP6K isoforms reduces VSMC calcification (Figure 10). Pharmacological inhibition of IP6K has already been shown to inhibit VC in rats with CKD, but this effect has been linked to a reduction in hyperphosphatemia [24]. It seems surprising that all three IP6K isoforms appear to promote VSMC calcification, even though they have diverging functional effects and expression profiles [28,29]. Although renal phosphate adaptation depends on IP6K1/2, IP6K3 has also been linked to serum phosphate concentrations by a genome-wide association study [23,30]. The highest expression for IP6K3 in humans was reported in muscle, thyroid, and heart, with only low abundance in the aorta [22]. But IP6K3 might still be of functional relevance in VSMCs and was found to be downregulated in the aortic wall after myocardial infarction [31]. For systematic evaluation, we included all three isoforms in our experimental design despite low expression levels of IP6K3 in aortic tissue. However, the current observations must be interpreted carefully, as an altered compensatory metabolic flux after gene silencing cannot be ruled out.

Nonetheless, the current results suggest that IP6K inhibition in VSMCs may induce an anti-calcific effect. During phosphate-induced VSMC calcification, cellular phosphate uptake through the phosphate transporter PiT1 or endocytosis of calciprotein particles may play an important role in the pro-calcific signaling [6,8]. These pathways induce cellular alterations, which then foster a pro-calcific microenvironment [8]. Inhibition of PiT1 or lysosomal calcium-phosphate processing impairs the calcification response of VSMCs [32,33,34,35]. Thus, the observed protective role of IP6K silencing seems somewhat surprising, as IP6K inhibition was linked to increased intracellular phosphate concentrations, apparently involving inhibition of phosphate export through XPR1 [20,24]. Although the exact functions, mechanisms, and regulations of XPR1 as a phosphate exporter are rather complex [36,37], mice heterozygous for XPR1 develop VC in the brain [38]. Knockout of XPR1 exacerbates calcification of human VSMCs [39]. Therefore, inhibition of XPR1-mediated cellular phosphate export does not seem a likely explanation for the anti-calcific effects of IP6K knockdown. However, the role of XPR1 in cellular phosphate homeostasis may also involve complex interactions. With the involvement of the inositol pyrophosphate IP8, organellar XPR1 regulates PiT1, which is also expressed in intracellular structures [40,41,42]. Although no profound effect on mRNA expression of *PiT1* (*SLC20A1*) or *XPR1* was observed, the interpretations of the current experiments are limited by the absence of phosphate transport measurements. An involvement of altered phosphate transport cannot be ruled out in the observed effects of IP6K silencing.

Since silencing of any of the three isoforms induces protective effects in calcifying VSMCs, it is tempting to speculate on a uniform effector pathway downstream of the IP6Ks. In an alternative attempt to link IP6K silencing effects to VC pathways, we investigated the involvement of AKT signaling. The IP6K product 5-IP7 could act as a potent inhibitor of AKT, via inhibition of AKT phosphorylation by phosphoinositide-dependent kinase-1 (PDK1) [25]. Accordingly, a pan-IP6K inhibitor ameliorates weight gain and insulin resistance in mice on a high-fat diet [43]. The inhibitory effects on AKT are best described for IP6K1, and cells from IP6K1-deficient mice respond with increased AKT phosphorylation at Thr^308^ upon IGF1 stimulation [25]. In dHL60 cells, overexpression of any IP6K isoform apparently interferes with AKT signaling [44]. It has been hypothesized that casein kinase-2 might modulate AKT signaling through IP6K2 de-stabilization [45]. IP6K2 is expressed mostly in the nucleus, but it can translocate to the cytoplasm upon cell stress and could contribute to IP7 production [46]. The current experiments focused on IP6K2, as in human vascular tissue, the IP6K2 might be the highest expressed isoform [22]. AKT exhibits a complex role in VSMC calcification [47]. While inhibition of its upstream phosphatidylinositol 3-kinase (PI3K) pathway has often been linked to anti-calcific effects in VSMCs [48,49], AKT1 silencing actually abrogated the protective effect of miR155 deficiency in calcifying VSMCs [50]. In the current experiments, the AKT inhibitor SH-6 tended to reduce the expression of *ALPL* in negative control siRNA-transfected VSMCs under calcifying conditions, but SH-6 actually abrogated the protective effects of IP6K2 silencing. Interestingly, similar effects of the SH-6 inhibitor were noted for the anti-calcific effects of farnesyl transferase inhibitor 227 [51].

In theory, the PI3K pathway could diverge between AKT and SGK1 [52], and AKT inhibition could tip the balance towards a potentially more pro-calcific SGK1 pathway [50]. SGK1 is dynamically regulated on transcriptional level and, like AKT, is also activated by PDK1 [53]. In accordance, a SGK1 inhibitor could counter the effects of the AKT inhibitor on *ALPL* mRNA expression during IP6K2 silencing. This hypothesis is further supported by endometrial cells, where the AKT inhibitor SH-6 apparently increases SGK1 activity [54]. However, the effects of the AKT pathway during VC appear rather complex and context-dependent [55]. The current experiments cannot rule out indirect regulations and other critical pathways, regulated by individual or all IP6K isoforms during VC. In all these observations, it must be kept in mind that cell culture models are highly dependent on culturing conditions and not directly translatable to in vivo conditions. Also, pharmacological inhibitors may have unspecific effects [56] and other effects and mechanisms cannot be ruled out.

In conclusion, silencing of any IP6K isoform in VSMCs reduces calcification during high phosphate conditions, effects possibly mediated by an altered balance between AKT and SGK1 signaling. Further experiments in vivo are required to establish a functional relevance for the current observations.

## 4. Materials and Methods

### 4.1. Cell Culture of Primary Human Aortic Smooth Muscle Cells (HAoSMCs)

HAoSMCs (Thermo Fisher Scientific, Vienna, Austria) were routinely cultured as previously described [34,57] and used in experiments up to passage 12. Where indicated, HAoSMCs were transfected with 10 nM IP6K1 (ID: s18957), IP6K2 (ID: s296), IP6K3 (ID: s42154), or negative control (ID: 4390843) siRNA using siPORT amine transfection reagent (all from Thermo Fisher Scientific, Vienna, Austria). HAoSMCs were treated for the indicated times with calcification medium supplemented with 10 mM β-glycerophosphate and 1.5 mM CaCl_2_ (Merck, Vienna, Austria) [58] and/or 10 µM AKT inhibitor SH-6 (stock in DMSO, Santa Cruz Biotechnology, Heidelberg, Germany) [51] or 50 µM SGK1 inhibitor EMD638683 (stock in DMSO, Biorbyt, Cambridge, UK) [59,60,61]. An equal amount of vehicle was used as a control. For long-term treatments, fresh medium with agents was added every 2–3 days.

### 4.2. RNA Isolation and RT-PCR

Total RNA was isolated from HAoSMCs by using Trizol Reagent (Thermo Fisher Scientific, Vienna, Austria). cDNA synthesis was performed by using SuperScript III Reverse Transcriptase and oligo(dT)_12–18_ primers (Thermo Fisher Scientific, Vienna, Austria). RT-PCR was performed in duplicate with iQ Sybr Green Supermix (Bio-Rad Laboratories, Vienna, Austria) and the following human primers (Thermo Fisher Scientific, Vienna, Austria) [27,62]:

*ALPL* fw: GGGACTGGTACTCAGACAACG;

*ALPL* rev: GTAGGCGATGTCCTTACAGCC;

*BGLAP* fw: CACTCCTCGCCCTATTGGC;

*BGLAP* rev: CCCTCCTGCTTGGACACAAAG;

*BMP2* fw: TTCGGCCTGAAACAGAGACC;

*BMP2* rev: CCTGAGTGCCTGCGATACAG;

*CBFA1* fw: GCCTTCCACTCTCAGTAAGAAGA;

*CBFA1* rev: GCCTGGGGTCTGAAAAAGGG;

*GAPDH* fw: GAGTCAACGGATTTGGTCGT;

*GAPDH* rev: GACAAGCTTCCCGTTCTCAG;

*IP6K1* fw: GAGTCCAAGGACCGAAAGCTC;

*IP6K1* rev: AACACGCAGGGGTACTTGAAG;

*IP6K2* fw: TAACCCTTGGAGCATGAAATGTC;

*IP6K2* rev: TCATAGCGGGAAGTCAGGTTT;

*IP6K3* fw: AACCAGGTTGAGAGGAAGAGC;

*IP6K3* rev: CGCTTGTTCTCTGGGTACTCG.

*MSX2* fw: TGCAGAGCGTGCAGAGTTC;

*MSX2* rev: GGCAGCATAGGTTTTGCAGC;

*SLC20A1* fw: GGAAGGGCTTGATTGACGTG;

*SLC20A1* rev: CAGAACCAAACATAGCACTGACT;

*SP7* fw: CACAAAGAAGCCGTACTCTGT;

*SP7* rev: GGGGCTGGATAAGCATCCC;

*XPR1* fw: GAGTGGCTCACGTAGAGGTG;

*XPR1* rev: ACGTAAACGCTTCATAGCCTTT.

Relative mRNA expression was calculated by the 2^−ΔΔCt^ method using GAPDH as the housekeeping gene.

### 4.3. Immunofluorescence Staining and Confocal Microscopy

HAoSMCs were fixed in 4% PFA/PBS for 15 min and permeabilized with 0.3% TritonX-100/PBS for 10 min. Slides were blocked with 5% goat serum in 0.1% TritonX-100/PBS for 1 h at RT. Cells were incubated with primary rabbit anti-RUNX2 antibody (1:100 dilution; 12556, Cell Signaling, Frankfurt am Main, Germany) [13,58] at 4 °C overnight and then with goat anti-rabbit Alexa488-conjugated antibody (1:500 dilution; Thermo Fisher Scientific, Vienna, Austria) for 2 h at RT. Nuclei were stained with DAPI (0.5 µg/mL; Thermo Fisher Scientific, Vienna, Austria) for 5 min at RT, and slides were mounted with Prolong Diamond antifade reagent (Thermo Fisher Scientific, Vienna, Austria). Images were acquired on a Nikon Ti-2 microscope (x60 oil immersion, NA 1.42, Nikon, Amstelveen, The Netherlands) equipped with a Clarity Laser Free Confocal Unit (Aurox, Abingdon, UK).

### 4.4. Protein Isolation and Western Blotting

Total proteins were isolated from HAoSMCs with ice-cold Pierce IP lysis buffer (Thermo Fisher Scientific, Vienna, Austria) supplemented with complete protease and phosphatase inhibitors cocktail (Thermo Fisher Scientific, Vienna, Austria), and protein concentrations were determined by the Bradford assay (Bio-Rad Laboratories, Vienna, Austria). Equal amounts of protein were boiled in Roti-Load1 Buffer (Carl Roth, Karlsruhe, Germany) at 100 °C for 10 min, separated on SDS-PAGE gels, and transferred to PVDF membranes (Roche Applied Science, Mannheim, Germany). Membranes were incubated with primary antibodies: rabbit anti-phospho-AKT (Thr^308^) (1:1000 dilution; 4056, Cell Signaling, Frankfurt am Main, Germany), rabbit anti-AKT (1:1000 dilution; 9272, Cell Signaling, Frankfurt am Main, Germany), or rabbit anti-GAPDH (1:3000 dilution; 2118, Cell Signaling, Frankfurt am Main, Germany) at 4 °C overnight and then with secondary anti-rabbit HRP-conjugated antibody (1:1000 dilution; Cell Signaling, Frankfurt am Main, Germany) for 1 h at RT. Membranes were stripped with Restore Plus Western blot stripping buffer (Thermo Fisher Scientific, Vienna, Austria) at RT. Bands were detected with Clarity Western ECL substrate (Bio-Rad Laboratories, Vienna, Austria) with the ChemiDoc MP imaging system (Bio-Rad Laboratories, Vienna, Austria) and quantified with the ImageJ software (NIH, Bethesda, MD, USA, 1.53e). Data are shown as the ratio of phosphorylated to total protein to GAPDH and of total protein to GAPDH, normalized to the control group [27,59,63].

### 4.5. Calcification Analysis

HAoSMCs were incubated overnight at 37 °C with OsteoSense 680EX (1:250, Revvity, Traiskirchen, Austria) and images were acquired with the ChemiDoc MP imaging system (Bio-Rad Laboratories, Vienna, Austria) [64]. HAoSMCs were decalcified overnight at 4 °C in 0.6 M HCl, and the quantification of calcium content was performed by using the QuantiChrom Calcium assay kit (BioAssay Systems, Hayward, CA, USA). Proteins were isolated by using 0.1 M NaOH/0.1% SDS buffer and quantified by the Bradford assay (Bio-Rad Laboratories, Vienna, Austria). Data are shown normalized to total protein concentration and to the control group [65].

### 4.6. Statistics

Data are presented as scatter dot plots and arithmetic means ± SEM, and *n* represents the number of independent experiments performed. Normalized data are shown as arbitrary units (a.u.). Normality was determined by the Shapiro–Wilk test. For two groups, statistical testing was performed using an unpaired *t*-test. For multiple group comparison, statistical testing was performed by using one-way ANOVA with Tukey’s test (homoscedastic data) or the Games–Howell test (heteroscedastic data) and the Kruskal–Wallis test with the Steel–Dwass test (non-normal data). *p* < 0.05 was considered statistically significant.

## Data Availability

The original contributions presented in the study are included in the article, and further inquiries can be directed to the corresponding author.

## Supplementary Materials

The following supporting information can be downloaded at: https://www.mdpi.com/article/10.3390/ijms27031411/s1.
