# Supplementary material for: Role of Inositol Hexakisphosphate Kinases in Vascular Smooth Muscle Cell Calcification
Source: Int J Mol Sci. 2026 Jan 30;27(3):1411. doi: 10.3390/ijms27031411 (PMC12897964; doi:10.3390/ijms27031411)

Role of inositol hexakisphosphate kinases in vascular smooth muscle cell calcification

Sheyda Bahiraii[1], Isratul Jannat[1], Sarah Plösser[1], Mehdi Razazian[1], Jakob Voelkl[1,2,3], Ioana Alesutan[1]

Supplemental Material

Supplemental Figures

Suppl. Figure S1. Effects of IP6K2 knockdown and AKT inhibition on phosphate transporter expression in VSMCs during calcifying conditions. Relative mRNA expression (n=8) of *XPR1* (**A**) and *SLC20A1* (**B**) in HAoSMCs transfected for 72h with negative control (Neg.si) or IP6K2 (IP6K2si) siRNA and treated for 48h with control or calcification medium (Calc.) without and with 10 µM AKT inhibitor SH-6. *(p<0.05) significant compared to Neg.si-transfected group.

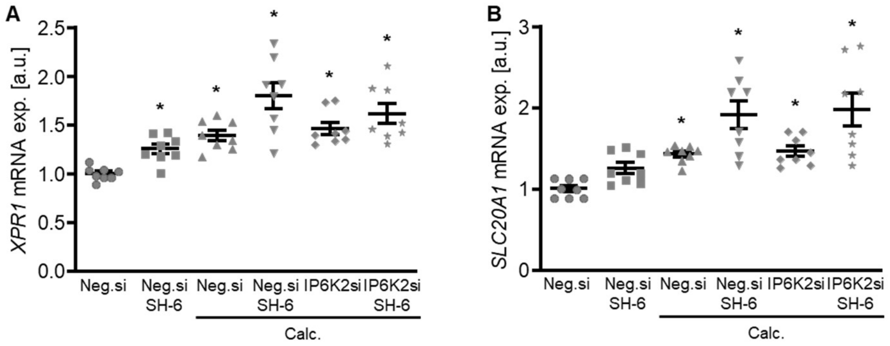

Supplement: Supplementary file 1 [file ijms-27-01411-s001.zip › ijms-3844756-supplementary.pdf]
